# Geometric and dosimetric consequences of intra-fractional movement in single isocenter non-coplanar stereotactic radiosurgery

**DOI:** 10.1186/s13014-022-02195-z

**Published:** 2023-01-11

**Authors:** Nelson Tsz Cheong Fung, Wai Lung Wong, Michael Chi Hang Lee, Elki Sze Nga Cheung, Philip Yuguang Wu

**Affiliations:** 1 Department of Medical Physics, Pamela Youde Nethersole Eastern Hospital, Hong Kong, China; 2 Department of Clinical Oncology, Pamela Youde Nethersole Eastern Hospital, Hong Kong, China

## Abstract

**Purpose:**

To investigate the geometric and dosimetric impacts of intra-fractional movement for patients with single or multiple brain metastasis treated using Varian Hyperarc™ mono-isocentric radiosurgery.

**Methods:**

A total of 50 single or hypo-fractionated Hyperarc™ treatment courses (118 lesions) were included in the analysis. Intra-fractional translational and rotational movements were quantified according to the post-treatment cone-beam CT (CBCT). Geometric displacements of all targets were calculated individually based on the assessed head movement in each treatment fraction and their relationships with treatment time and target-to-isocenter distances were studied. For dosimetric analysis, only single-fraction treatments (56 lesions) were included. Re-planning was performed with 0, 1, and 2 mm planning target volume (PTV) margins. Doses were then re-calculated on rotated CT images with isocenter shifted which emulate the change in patient treatment position. Target coverage, target and normal brain doses before and after intra-fractional movement were compared.

**Results:**

The mean 3D target displacements was 0.6 ± 0.3 (SD) mm. Target shifts for patients treated within 10 min were significantly smaller than those treated in longer sessions. No correlation was found between target shift and target-to-isocenter distance as the origin of head rotation was not located at the isocenter. Loss of target coverage and minimum Gross Tumor Volume (GTV) dose due to intra-fractional movement were apparent only when no margin was used, leading to an extra 23% of the targets violating the dose acceptance criteria, in contrast, the effects on normal brain *V*_12Gy_ were negligible regardless of the margin used. The use of 1 mm PTV margin can compensate clinically significant geographical miss caused by intra-fractional movements while limiting *V*_12Gy_ to within dose criteria for 88% of the cases. The plan acceptance rate (fulfillment of both target and normal brain dose criteria) after intra-fractional movement was also the highest with the 1 mm margin.

**Conclusion:**

Although intra-fractional movements during Hyperarc™ treatments were small, there were substantial dosimetric effects due to the sharp dose fall-off near target boundaries. These effects could be mitigated by using a 1 mm PTV margin and maintaining the effective treatment time to within 10 min.

## Introduction

Approximately 20–40% of patients with advanced cancers develop brain metastasis (BM) [1] and the clinical demand has increased in recent years due to increased sensitivity of radiological diagnosis and improved overall survival resulting from evolving systemic treatment options for advanced cancers [2]. Stereotactic radiosurgery (SRS) has been shown to be an effective and safe treatment for single or multiple BMs [3, 4]. Early data on SRS was largely limited to patients with up to 4 BMs. With emerging evidence from multi-institutional studies [5, 6], it is increasingly reassuring that SRS is an effective treatment for patients with extensive BMs (5 or more lesions), providing good local control and similar overall survival and superior neurocognitive preservation and quality of life compared to whole brain radiation therapy (WBRT), which has been the historical standard of care.

Conventional linac-based SRS treatments are typically performed using the multi-isocenter approach, i.e. each lesion is treated in a one-by-one manner with separate isocenter position. Multiple episodes of setup and beam-on were required for treatment of multiple BMs which can be exceedingly time consuming and physically demanding for patients. The single isocenter multi-target approach (SIMT) was therefore proposed [7] to speed up the treatment delivery process by allowing the simultaneous off-axis irradiation of multiple lesions without having to change the isocenter position. Recently, various commercial solutions have become available, one of the mostly widely used packages is Hyperarc™ (Varian Medical Systems, Palo Alto, CA)—a software that supports automatic optimization and delivery of non-coplanar, multi-leaf collimator (MLC)-based SIMT SRS treatments.

SRS treatment calls for extremely high accuracy in treatment delivery, and many advancements in technique and technology are developed to reduce the positional uncertainty/discrepancy resulted from machine in-precision, target localization error, setup error, intra-fractional patient movements, etc. During Hyperarc™ treatments, patient’s treatment position is fixated using the Encompass™ frameless-based immobilization system. Such frameless approach provides good patient comfort, allows reproducible setup for fractionated treatments and is completely non-invasive. Nevertheless, there is potential of intra-fractional movements which may result in geometrical miss to the target lesion and unintended dose exposure to adjacent normal tissues.

This paper investigated the geometrical and dosimetric impact of intra-fractional movements in Hyperarc™ SRS. In particular, we would like to fill in the current gap in the available literature on the following areas:

1. Most intra-fractional movements studies [8–12] investigated the overall head movement in terms of translation and rotation about the isocenter. However, how such movement translates into individual tumor shifts remains uncertain, particularly for targets that are distant from the isocenter in SIMT SRS treatments.
2. Some studies [10, 13, 14], proposed that tumor shifts due to intra-fractional rotation could increase with the target-to-isocenter distance. This would be true if the origin of the head rotation is located at the isocenter, yet it is unclear how the two could be related physically. Thus, it is questionable whether the target-to-isocenter distance has any effect on tumor shifts as far as intra-fractional movements are concerned.
3. To quantify the loss in target coverage and the dosimetric impact on normal brain tissues, and to find the PTV margin required for mitigating dose distribution degradation caused by intra-fractional movements.

## Materials and methods

### Planning and treatment data

This study was reviewed and approved by the Hong Kong East Cluster Research Ethics Committee, Hospital Authority. Consecutive patients with single or multiple brain metastasis treated using Varian Hyperarc™ stereotactic radiosurgery in Pamela Youde Nethersole Eastern Hospital, Hong Kong between February 2020 to March 2022 were recruited retrospectively for the study, excluding cases that received both single and multi-fractionated treatments in the same treatment course. Treatment was planned using the Hyperarc™ module available in the Eclipse™ treatment planning system (version 15.5, Varian Medical System) for delivery on a dedicated Varian Truebeam™ Linac using 6MV flattening filter free (FFF) beams.

The QFix Encompass™, a clam-shell style mask-based immobilization system was used. It consists of an anterior and a posterior portion which could be locked together with multiple pins, while the posterior part is attached to the Encompass overlay board. Adjustable shims can be used to maintain the tightness of the mask during treatment and bite blocks were used whenever possible to reduce intra-fractional movements.

Pre-treatment setup Cone beam CTs (CBCTs) were used to align the patients as planned at zero couch angle. After the treatment delivery, post-treatment CBCTs were taken to assess the translational and rotational intra-fractional movements. The amplitudes of the movements were measured by online matching the post-treatment CBCT with the planning CT (pCT) using auto-registration in six degrees of freedom. The same configuration (including the volume of interest and intensity range) was used for both pre- and post- CBCT-to-pCT matching. For all cases, no patient setup correction was made between the pre- and post- CBCT.

### Geometric analysis

Under the Varian™ IEC coordinate system, and applying approximation of small rotational angles, the individual target shift $\overrightarrow{\Delta r}$ for a particular GTV can be estimated as (see Appendix for more detail):

1 $$\overrightarrow{\Delta r}=\left(\begin{array}{c}\Delta x\\ \Delta y\\ \Delta z\end{array}\right)\approx \left(\begin{array}{ccc}0& \varphi & -\psi \\ -\varphi & 0& \theta \\ \psi & -\theta & 0\end{array}\right)\cdot \overrightarrow{{\mathrm{r}}_{\mathrm{CT}}}-\left(\begin{array}{c}\mathrm{Lat}\\ \mathrm{Vrt}\\ \mathrm{Lng}\end{array}\right),$$

where $\psi$, $\theta$, $\varphi$, Vrt, Lng, Lat are the online match readings of the yaw, pitch and roll in radians, as well as the vertical, longitudinal and lateral translation shifts respectively. $\overrightarrow{{\mathrm{r}}_{\mathrm{CT}}}$ is the coordinate of the center of mass of the GTV relative to the isocenter specified in the pCT, while $\Delta x$, $\Delta y$ and $\Delta z$ represent the target shifts in the left–right, posterior-anterior, and superior-inferior directions respectively. The 3D displacement ${\Delta }_{\mathrm{tot}}$ is defined as the total magnitude of the target shift, i.e.

2 $${\Delta }_{\mathrm{tot}}={({\Delta x}^{2}+{\Delta y}^{2}+{\Delta z}^{2})}^\frac{1}{2}$$

Correlation of the magnitudes of individual target shifts and treatment time (defined as the difference between the acquisition time of the pre- and post- treatment CBCTs) were tested using one-tailed Spearman’s rank test. Difference of target shift magnitudes for treatments that is less than 10 min and greater than or equal to 10 min were tested using the one-tail Mann–Whitney U test.

Similar to treatment time, correlation of target shifts with target-to-isocenter distances was also tested using the one-tailed Spearman’s rank test. This test, however, could be potentially biased by the amplitudes of rotational angles which are covariates that could affect the target shifts. To mitigate such effect, the partial Spearman’s correlation test was performed with control on the variable angle_RMS_, where angle_RMS_ is the root mean square of all the rotation angles, i.e.

3 $${\mathrm{angle}}_{\mathrm{RMS}}={\left({\psi }^{2}+{\theta }^{2}+{\varphi }^{2}\right)}^\frac{1}{2}.$$

All correlation tests were executed on IBM SPSS statistics version 22, and *p* < 0.05 was considered statistically significant.

### Dosimetric analysis

While the geometrical study included both single and multi-fraction cases, only single-fraction SRS cases were included for the dosimetric analysis. We did not include fractionated cases for dosimetric analysis because they were planned with a different dosimetric criteria, and often involved heterogeneous target types (e.g. large surgical cavity).

The impact on target and normal brain doses were examined for 3 different PTV margins (0 mm, 1 mm, 2 mm). Three different study plans were created for each treatment course, one for each margin. Target prescription doses of the study plans were the same as those in the clinical plans (24 Gy for targets less than or equal to 4.2 cc, 18 Gy for targets between 4.3 cc and 14.1 cc; fractionated treatments were given to surgical cavity or targets that are larger than 14.1 cc and so these cases were not included in the dosimetric analysis). The optimization was set to achieve a 99% PTV coverage by the prescription dose, without compromising dose tolerance of critical organs (e.g. brainstem).

To simulate individual patient’s intra-fractional movements, the pCTs were first rotated using the ‘Rotate view’ tool available in the MIM 6.3 (MIM software Inc) according to the online match readings (post-CBCT to pCT) of each treatment. The rotated CT images were then sent to Eclipse where rigid registration with the pCT was performed. After registration, patient contours were propagated from the pCT to the rotated CT. Translational movements was simulated by shifting the isocenter position. The dose distribution was then re-calculated using the same treatment parameters and calculation settings (calculation algorithm: Acuros 15.5, dose grid size: 1.25 mm) and compared with that of the original plans. The changes in *V*_100%_, *V*_95%_ and *D*_min_ of the GTVs as well as *V*_12Gy_ of the normal brain tissue due to intra-fractional movements were examined. The increase in normal brain *V*_12Gy_ caused by PTV margin expansion were also evaluated. Wilcoxon signed rank tests (IBM SPSS statistics (version 22)) were performed to assess the changes, with *p* < 0.05 taken as statistically significant. Finally, the fulfillment of dose acceptance criteria before and after intra-fractional movements using different PTV margins were analyzed and compared. The dose acceptance criteria [15–18] were as follow: (1) Target coverage 1 (TC_1_): GTV volume receiving 100% prescription dose *V*_100%_ > 95%, (2) Target coverage 2 (TC_2_): GTV volume receiving 95% prescription dose *V*_95%_ > 99%, (3) Minimum GTV dose: *D*_min_ > 90% prescription dose, 4) Normal brain (Brain-GTV(s)) volume receiving 12 Gy *V*_12Gy_ < 10 cc. *V*_12Gy_ was determined for each individual target volume (i.e. isolated 12 Gy isodose volume of normal brain tissues adjacent to the individual target) except in cases when the 12 Gy isodose volumes of multiple targets bridged and formed a single contiguous volume.

Dose changes for other critical structures such as brainstem, optic apparatus, were not studied as most of them were far away from the target lesions (except for two cases in which the lesion was near the brainstem) and their dose levels were negligible.

## Results

### Patients and volume characteristics

A total of 50 consecutive patient cases were recruited and the details for their geometrical and dosimetric characteristics are summarized in Table 1. There were a total of 110 fractions (amounted to 244 data points) for the target shift evaluation. 21 patients were treated with single fraction SRS: between them there were 56 GTV’s and 49 normal brain *V*_12Gy_ volumes for the dosimetric analysis; 3 GTV pairs and 2 Tri-GTV sets were so close together they constituted to 5 *V*_12Gy_ volumes. The characteristics of the target volumes and dose quality indexes of the 63 study plans created with different margins (before intra-fractional movements) can be found in the Appendix (Table 5).

**Table 1:** Patient data used for geometric and dosimetric analysis

Description	Geometric analysis (*n*)	Dosimetric analysis (*n*)
*Patient cases*		
Solitary lesion	18	5
Multiple lesions	32	16
Total	50	21
*Fractions*		
1 fraction	21	21
3 fractions	28	0
5 fractions	1	0
Total	110	21
*Targets*		
Isocentric	18	5
Off-axis	100	51
Total	118	56
*Target prescription*		
24 Gy/1 fr	NA	48
18 Gy/1 fr	NA	8
Total	NA	56
*Data points*		
Targets	244	56
Normal brain	NA	49

### Translational and rotational head movements

Table 2 shows the statistics of translational and rotational (about the isocenter) intra-fractional movements of the head. The magnitudes of the shifts detected in the lateral, vertical, longitudinal, yaw, pitch and roll directions were (mean ± SD) 0.3 ± 0.2 mm, 0.2 ± 0.2 mm, 0.3 ± 0.3 mm, 0.2 ± 0.3°, 0.2 ± 0.3° and 0.2 ± 0.2°, respectively. Both the mean translation and rotation (without taking absolute values) were close to zero and small compared to the standard deviation (SD) indicating that there was negligible systematic error introduced by intra-fraction movements. For translation, the SD, maximum, 90th and 95th percentiles of the longitudinal shifts were the largest than other two directions while for rotation, the SD, 90th and 95th percentiles of the shifts were similar in all directions, although one outlier case with a large pitch of 2.3° was observed.

**Table 2:** Statistics of online match readings and individual target shifts

	Online match readings						Individual target shifts			
	Lat (mm)	Vrt (mm)	Lng (mm)	Yaw (°)	Pitch (°)	Roll (°)	Δx (mm)	Δy (mm)	Δz (mm)	${\Delta }_{\mathrm{tot}}$(mm)
Mean	− 0.2	− 0.1	0.0	0.0	0.1	0.0	0.2	0.1	0.0	0.6
SD	0.3	0.2	0.4	0.4	0.4	0.3	0.3	0.3	0.4	0.3
Mean (abs)	0.3	0.2	0.3	0.2	0.2	0.2	0.3	0.2	0.3	0.6
SD (abs)	0.2	0.2	0.3	0.3	0.3	0.2	0.3	0.3	0.4	0.3
Max (abs)	1.1	0.8	1.9	1.3	2.3	1.0	1.1	0.8	2.0	2.2
90^th^ percentile (abs)	0.6	0.4	0.8	0.6	0.6	0.5	0.7	0.4	0.7	1.0
95^th^ percentile (abs)	0.7	0.5	1.0	0.8	0.6	0.7	0.7	0.6	0.9	1.2

Online match readings were obtained by registering the post-treatment CBCT to the planning CT. Individual target shifts were calculated based on the acquired readings which quantified the intra-fractional movement during Hyperarc™ treatments. Here, abs and SD stand for the abbreviations for absolute values and standard deviations respectively

### Individual 3D target displacements

Statistics of individual target shifts are also displayed in Table 2. The total target displacements ${\Delta }_{\mathrm{tot}}$ in 3D space was 0.6 ± 0.3 mm (SD) and 91% of them were less than 1 mm. The maximum ${\Delta }_{\mathrm{tot}}$ was 2.2 mm, this was the only time ${\Delta }_{\mathrm{tot}}$ was above 2 mm out of 244 data points. The 90th and 95th percentiles of ${\Delta }_{\mathrm{tot}}$ were 1.0 mm and 1.2 mm respectively. Similar to the situation with the whole head, the mean shifts were also very close to zero, and the SD, maximum, and 95th percentile of the shifts were the largest in the superior-inferior direction (Δz).

### Time dependency of target shifts

**Fig. 1:**
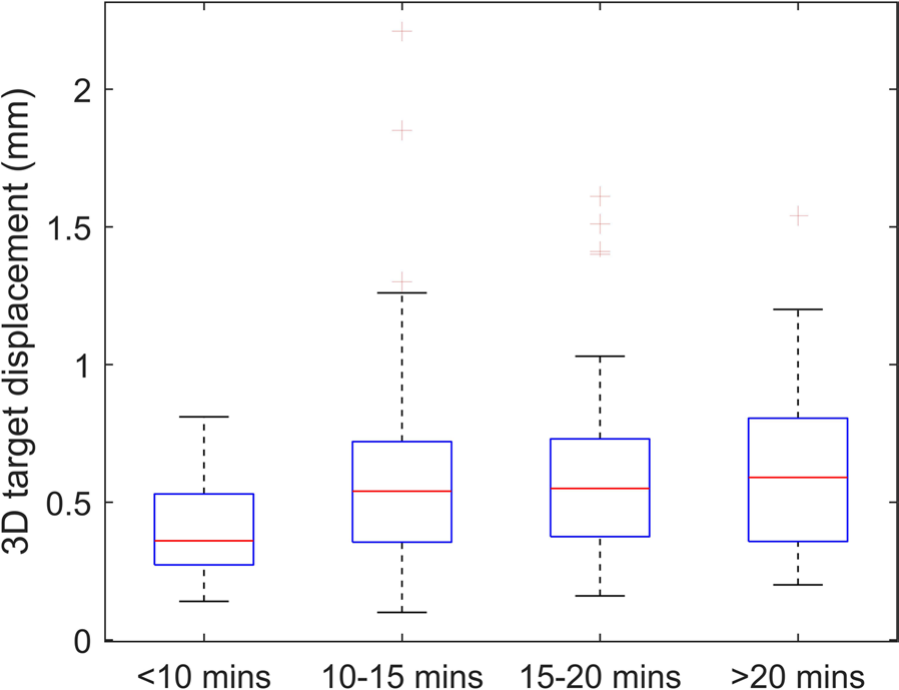
Statistics of individual target shifts for 4 different treatment time groups (< 10, 10–15, 15–20 and > 20 min)

Figure 1 shows the statistics of individual 3D displacements for four treatment time groups (< 10, 10–15, 15–20 and > 20 min). The one-tailed Spearman’s test (*n* = 244) resulted in a correlation coefficient $\rho$ of 0.159, which corresponds to a *p*-value of 0.006. Hence, there was a statistically significant correlation between treatment time and target shifts. Although a systematic increase in ${\Delta }_{\mathrm{tot}}$ was observed with increasing treatment time, increase of target displacements tended to level off when treatment time was larger than 10 min. Besides, when the treatment time was kept under 10 min, none of the targets exceeded 1 mm ${\Delta }_{\mathrm{tot}}$. The one-tailed Mann–Whitney U test also concluded a significant difference (*p* = 0.001) in ${\Delta }_{\mathrm{tot}}$ between treatments with treatment time less than and larger than 10 min.

### Relationship of individual target shift with target-to-isocenter distance

**Fig. 2:**
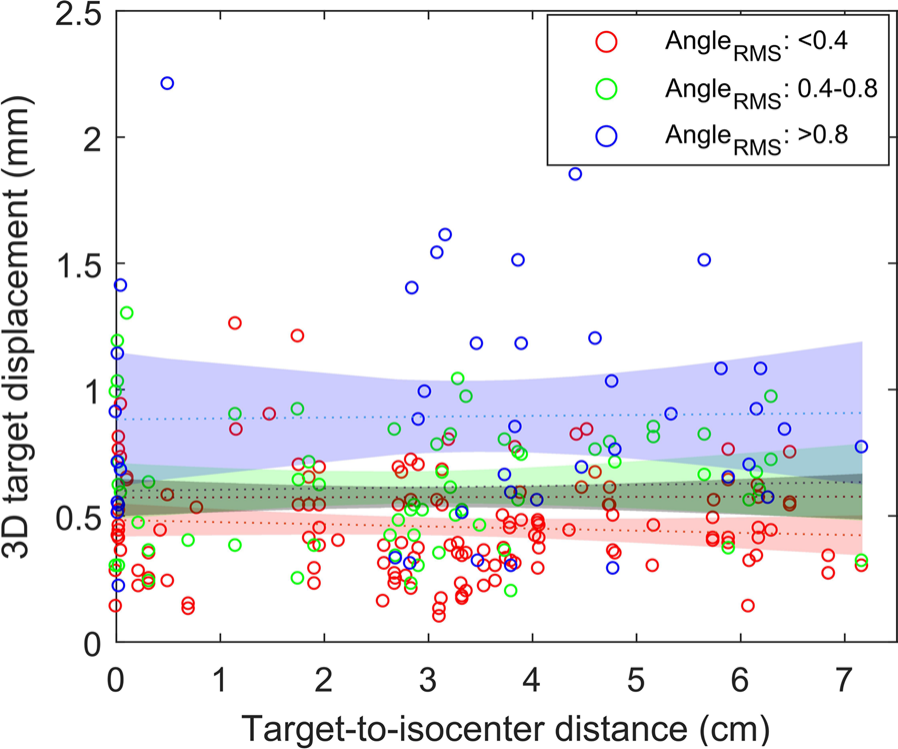
Plots of 3D target displacements against target-to-isocenter distances. Lines of best fit (linear regression) and confidence intervals for all data (grey), data with Angle_RMS_ < 0.4° (red), 0.4° ≤ Angle_RMS_ ≤ 0.8° (green) and Angle_RMS_ > 0.8° (blue) are displayed

The individual 3D target displacement ${\Delta }_{\mathrm{tot}}$ was plotted against the target-to-isocenter distance in Fig. 2. Linear regressions were performed for four data groups with Angle_RMS_ < 0.4°, 0.4° ≤ Angle_RMS_ ≤ 0.8°, Angle_RMS_ > 0.8°, and all data. The dotted line represents the line of best fit while the shaded area indicates the 95% confident bounds of the best fit. The Spearman’s rank correlation test (*n* = 244) showed that there is no significant correlation between ${\Delta }_{\mathrm{tot}}$ and target-to-isocenter distance (*p* = 0.170). No particular target-to-isocenter distance dependence can be observed for all of the groups, whereas there was an increasing trend of ${\Delta }_{\mathrm{tot}}$ with increasing Angle_RMS._ The partial correlation test (*n* = 244) also showed no association between ${\Delta }_{\mathrm{tot}}$ and target-to-isocenter distance, whilst controlling for Angle_RMS_ (*p* = 0.241).

### Loss of target coverage and minimum GTV dose reduction

Table 3 shows the percentage fulfillment of dose acceptance criteria in the original plan and in the simulated plan with intra-fractional movements. All three target dose criteria were met in all the original plans except for two targets due to its proximity to the brainstem.

**Table 3:** Percentage fulfillment of dose acceptance criteria before and after intra-fractional movement

PTV margin	TC_1_ (*V*_100%PD_ > 95%)	TC_2_ (*V*_95%PD_ > 99%)	*D*_min_ > 90% (GTV)	TC_1_ /TC_2_/*D*_min_ > 90%	V_12Gy_ < 10 cc (Normal brain)	All criteria
*Original plan*						
0 mm	98.2%	98.2%	96.4%	96.4%	98.0%	93.9%
1 mm	98.2%	98.2%	98.2%	98.2%	87.8%	85.7%
2 mm	98.2%	98.2%	98.2%	98.2%	71.4%	69.4%
*Simulated plan with intra-fraction movements*						
0 mm	85.7% (*p* < 0.001)	78.6% (*p* < 0.001)	76.8% (*p* < 0.001)	73.2% (NA)	98.0% (See Table 4)	69.4% (NA)
1 mm	98.2% (*p* = 0.07)	98.2% (*p* = 0.66)	98.2% (*p* < 0.001)	98.2% (NA)	87.8% (See Table 4)	85.7% (NA)
2 mm	98.2% (*p* = 0.18)	98.2% (*p* = 0.16)	98.2% (*p* < 0.001)	98.2% (NA)	71.4% (See Table 4)	69.4% (NA)

The bracketed data show the *p*-values evaluated for the changes of GTV *V*_100%PD_, *V*_95%PD_ and *D*_min_ before and after intra-fractional movements using the Wilcoxon signed rank tests

**Fig. 3:**
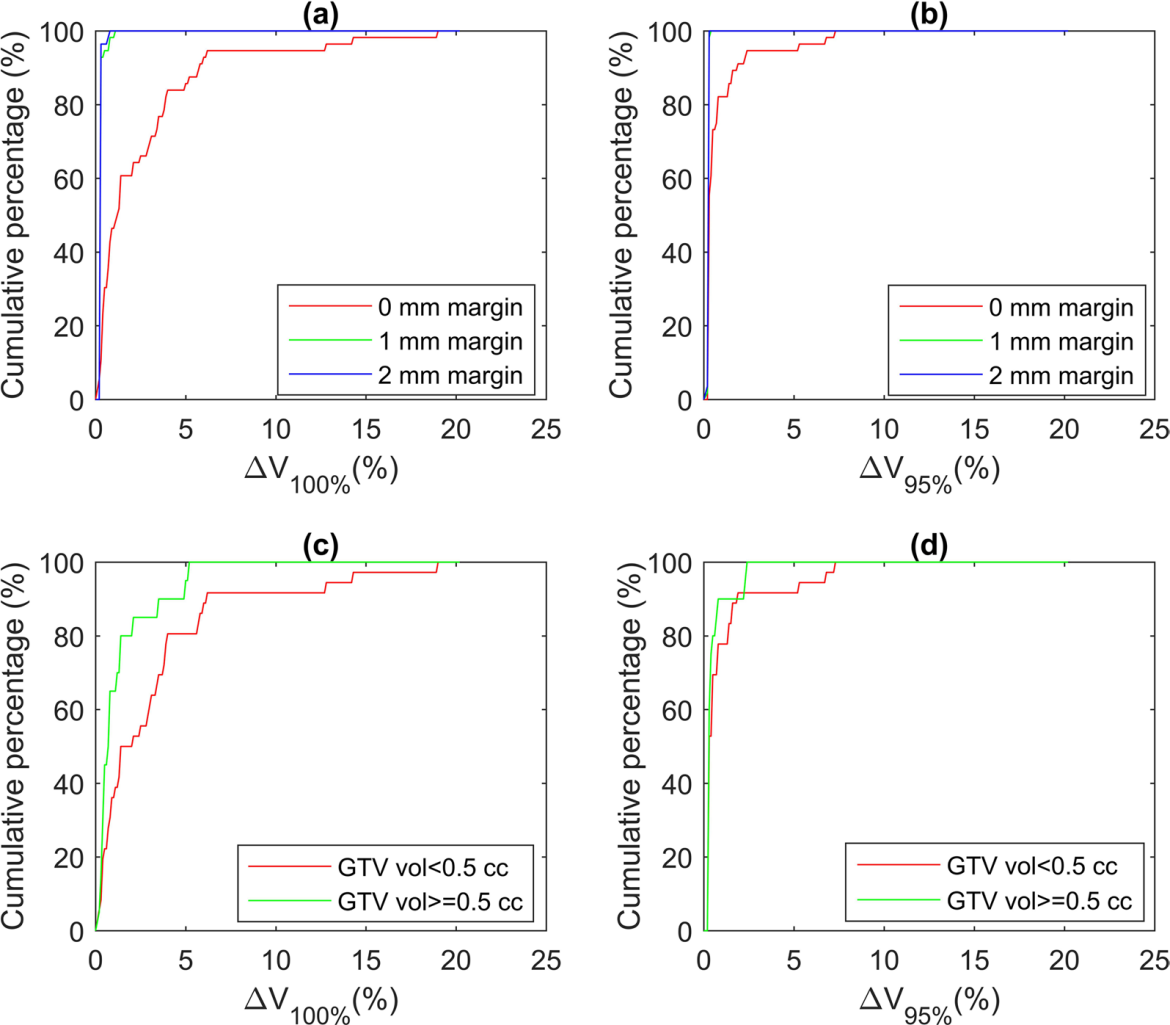
Cumulative percentages of cases with target coverage changes less than a certain percentage: Δ*V*_100%_ (**a**) and Δ*V*_95%_ (**b**) for 0, 1 and 2 mm PTV margin, Δ*V*_100%_ (**c**) and Δ*V*_95%_ (**d**) for GTV volume < 0.5 cc and GTV volume ≥ 0.5 cc when 0 mm PTV margin is used

As depicted in Fig. 3a and b, after target shifting, the reduction in target coverage in terms of *V*_100%_ and *V*_95%_ were minimal (less than 1% and 0.2% respectively) and did not reach statistical significance when 1 or 2 mm PTV margin was applied. Their changes were only significant (*p* < 0.001) when no PTV margin was used, particularly for smaller lesions. This is illustrated in Fig. 3c and d where the reductions in *V*_100%_ and *V*_95%_ were less than 5% and 3% (in absolute term) respectively when GTV was larger than or equal to 0.5 cc, while for smaller GTV these reductions were approximately 20% and 8% respectively.

**Fig. 4:**
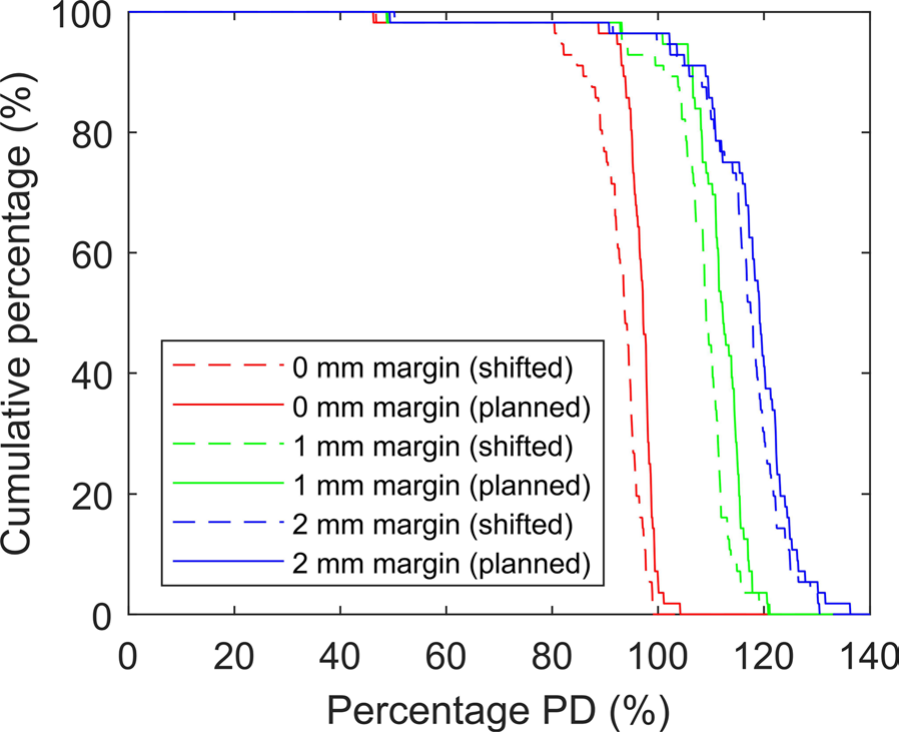
Cumulative percentages of cases with *D*_min_ larger than a certain percentage prescription dose (PD) for plans created with 0 mm, 1 mm and 2 mm PTV margins (Dose statistics for planned (solid line) and shifted (dashed line) targets were compared). Optimization was set to achieve a 99% PTV coverage by the prescription dose without compromising dose tolerance of critical organs

Intra-fractional movements could also lead to a drop of *D*_min_ of the GTV (*p* < 0.001 for 0, 1 or 2 mm margin). Figure 4 compared the *D*_min_ before and after intra-fractional movements. The mean reduction in *D*_min_ (in % of prescribed dose) was slightly higher when no PTV margin was used (4.0% as compared to 3.2% and 1.5% for 1 and 2 mm margins respectively). The number of targets with *D*_min_ > 90% prescription dose (PD) had dropped significantly from 54 (96.4%) to 43 (76.8%). By adding a 1 mm PTV margin, the number of targets with GTV *D*_min_ > 90% PD would remain unchanged after intra-fractional movements.

In fact, the number of targets fulfilling all the target dose criteria was only affected by intra-fractional movements when no PTV margin was used, in which case an extra 23.2% of targets would fail at least one of the target dose criteria as a result of intra-fractional movements.

### Changes in normal brain doses

The change in *V*_12Gy_ due to intra-fractional movements were minimal (on average 0.0 cc regardless of the margin used) and only reached statistical significance (*p* = 0.04) in the cases of 1 mm PTV margin, for which it was clinically negligible.

Shown in Table 4 is the increase of *V*_12Gy_ caused by PTV margin expansion. This time Δ*V*_12Gy_ increased significantly (*p* < 0.001) by 1.2 cc and 2.4 cc respectively when 1 mm and 2 mm margins were employed. It also increased with the target volume and the maximum Δ*V*_12Gy_ was as high as 12.7 cc for a group of closely spaced lesions with a total volume of 15 cc. PTV margin was most dominant in affecting normal brain *V*_12Gy_, the percentage of plans that fulfilled the *V*_12Gy_ criteria decreased from 98 to 87.8% and 71.4% with the addition of 1 mm and 2 mm margin respectively (Table 3), with and without intra-fractional movements.

**Table 4:** Change in *V*_12Gy_ the normal brain (brain-GTV) due to intra-fractional movement or addition of PTV margin

Δ*V*_12Gy_ (cc)	Intra-fractional movement			Addition of PTV margin (compared to the plan without margin)		
	Mean	Max	*p*	Mean	Max	*p*
0 mm margin	0.0	0.1	0.09	NA	NA	< 0.001
1 mm margin	0.0	0.2	0.04	1.2	5.8	< 0.001
2 mm margin	0.0	0.1	0.10	2.4	12.7	< 0.001

### Overall dose distribution quality

The numbers of treatment plans fulfilling all the dose acceptance criteria (target coverage and normal brain sparing) before and after intra-fractional movements with different PTV margins are shown in Table 3. Before considering intra-fractional movement, the plans without PTV margin were the best among the three, with 46 (93.9%) cases (the 5 sets of close-together-GTV’s considered as 5 GTV’s) fulfilling all the dosimetric criteria. This was followed by the plans with 1 mm PTV margin (85.7%) and 2 mm PTV margin (69.4%).

However, once the intra-fractional movements were considered, the plan acceptance rate markedly decreased to 69.4% when no PTV margin was used, which is the same as that achieved by adding a 2 mm PTV margin. The plan acceptance rate with 1 mm PTV margin remained unchanged after intra-fractional movements and became the highest among the three at 85.7%.

## Discussion

In this study, sub-millimeter and sub-degree intra-fractional head movements can be achieved for patients immobilized using the Q-fix Encompass™ system. The standard deviation of the translational and rotational movements in all directions were 0.2–0.3 mm and 0.2°–0.3° respectively which were comparable with those reported in previous literature [9, 13, 19]. Such consistency indicates the robustness of the Encompass™ immobilization system and only minimal intra-fractional movements should be expected if the mask were fitted with proper techniques. Treatment time is another factor that may influence the magnitude of intra-fractional movements and we observed a significant increase in tumor shift with treatment time, consistent with Mangesius et al. [11] and Amelio et al. [20] despite the use of different mask systems. Minimizing the treatment duration could limit the extent of intra-fractional movements, and target shifts were noted to be below 1 mm for all of our studied cases treated within a 10-min period.

Various studies [8, 13, 14] suggest that individual tumor shifts due to intra-fractional movements would increase with target-to-isocenter distances, yet in this study, no correlation was found between the two. Universally, shifts due to rotational error increases with the distance to the rotational axis. Therefore, it is true that rotational errors originated about the isocenter (e.g. setup error due to uncertainties in online matching, mechanical uncertainties in gantry, collimator or couch angles, or misalignment of the imaging system) could amplify target shifts with increasing target-to-isocenter distance. However, head movements arise from movements about the joints at the neck, rotation about the patient longitudinal axis, facial muscles or perhaps movement of the jaw, all of which are independent of the isocenter position. Thus, target shifts introduced by intra-fractional patient movements alone should be independent of the target-to-isocenter distance.

Guckenberger et al. [21] have investigated the dosimetric effect of intra-fractional movements for SRS treatments using the traditional multi-isocentric technique. Similar to this study, they also concluded that adding a 1 mm PTV margin could avoid clinically significant loss of target coverage of due to intra-fractional movements. Their computed target coverage loss was even slighter worse than those in our patient cohort. This is likely to be the result of higher 3D target displacements in their study (0.9 mm).

The loss of percentage GTV coverage due to intra-fractional movements was more prominent for smaller lesions when no margin is used as the intra-fractional shift itself would constitute a higher proportion of the lesion’s dimension as the target volume decreases. It was also found that reduction in GTV *D*_min_ increased with decreasing PTV margins due to the sharp dose fall-off outside the PTV surface. On the other hand, the change in normal brain *V*_12Gy_ due to intra-fractional movements were negligible regardless of the margin. This was expected as minute head rotation and translation inside the head mask could only cause small changes in the beam path lengths and the subsequent combined effects on the dose volumes, the shape and thus the *V*_12Gy_ isodose volume had basically remained unchanged.

Addition of PTV margin to account for intra- fractional movements is advisable judging from the fact that loss of target coverage could be substantial when no margin is used (an extra 23% of the patients would fail to achieve at least one of the target dose criteria in our study (Table 3)). Nevertheless, it should be noted that such margin expansion would also increase *V*_12Gy_ of the normal brain, and as a result, more patients would have exceeded the *V*_12Gy_ < 10 cc criteria and may require fractionated treatments or lower dose prescription. Overall, the usage of a 1 mm margin provides the best compromise between the reduction of target miss due to intra-fractional movements and minimization of normal brain doses. 85.7% of the cases in our study fulfilled all dose acceptance criteria (both target coverage and brain sparing) after shifts induced by intra-fractional movements were accounted, as opposed to 69.4% when 0 mm or 2 mm margin was employed.

In this study, patient positions were only recorded using CBCT before and after the treatment, and potential movement during treatment was unknown. As the patient position would gradually drift away from the nominal position with time, comparing the baseline and end-of-treatment position would yield a worst case estimate of the intra-fractional movements induced by patients, thus the geometrical and dosimetric effects investigated are likely to be overestimated. One should however note that intra-fractional movement is only one source of uncertainty in SRS treatments, and other factors may arise from setup image registration, contouring, MRI distortions, and linac accuracies. These uncertainties were outside the scope of this current study. Increase in hotspot doses could be risky if a serial critical organ (such as the brainstem or optic apparatus) is in vicinity of the target lesion. Due to the limited number of such cases (only 2 out of 56 targets in our cohort were close to the brainstem), the dosimetric effects of these organ at risks could not be investigated in this study. Caution must be exercised when designing the PTV margin for treatment planning in such scenario. Finally, fractionated treatment could potentially reduce the dosimetric impact of intra-fractional movements as random movements tend to cancel out each other, such effect should be investigated in future studies.

## Conclusion

In this study, geometrical and dosimetric impacts of intra-fractional movements in Hyperarc™ mono-isocentric SRS treatment were examined. Our results showed that robust and stable treatment setup could be achieved using the proprietary Encompass immobilization system and over 90% of the intra-fractional target shifts were less than 1 mm. Target shifts were found to increase with treatment time, and shifts were observed to be sub-millimeter if treated within a 10-min duration. No correlation was found between intra-fractional target shifts and target-to-isocenter distance as the origin of head rotation was not located at the isocenter. Target coverage and minimum GTV doses could be compromised if no PTV margin was used, yet the changes in *V*_12Gy_ for normal brain were negligible regardless of the margin used. The addition of a 1 mm PTV margin could avoid target misses caused by intra-fractional movements while the increase in the planned normal brain *V*_12Gy_ were tolerable for the majority of cases. The highest rates of dose acceptance criteria fulfillment after intra-fractional movements were observed with 1 mm PTV margin. Strategies including the use of 1 mm PTV margin and treatment time minimization could be employed to mitigate the adverse effects of intra-fractional movements in Hyperarc™ SRS treatments.

## Figures and Tables

**Fig. 5:**
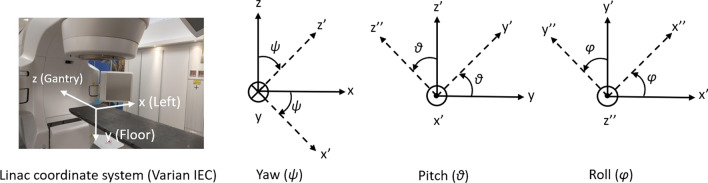
Illustration of rotational transformations between the linac coordinate system (Varian IEC 60201) and the patient support coordinate system

## Data Availability

The datasets used in the current study are available from the corresponding author upon reasonable request.

## Appendix

### A: Deviation of the target shift calculation formula

In the linac coordinate system (VarianIEC 60201), the x-axis is towards the left when facing the gantry, the y-axis is towards the treatment room floor, the z-axis is the direction towards the gantry and the system origin is defined at the isocenter. The isocentric standard couch representation is used, i.e. all rotation operation take place around linac’s isocenter (Fig. 5). Sequences of intrinsic rotations and translations are performed as follows to match the CBCT to the pCT [23]:

1. Yaw around the y-axis by the angle ${\psi}$
2. Pitch around the rotated patient support axis x′ by the angle ${\theta}$
3. Roll around the rotated and pitched patient support axis z″ by the angle $\boldsymbol{\varphi }$
4. Translation by Lat, Lng, and Vrt along the rotated, pitched and rolled patient support axis in the x″, y″ and z″ directions respectively.

We denote the coordinates of the center of mass of the GTV relative to the isocenter specified in the pCT and CBCT in the linac coordinate system (x, y, z) as $\overrightarrow{{\mathrm{r}}_{\mathrm{CT}}}$ and $\overrightarrow{{\mathrm{r}}_{\mathrm{CBCT}}}$ respectively. $\overrightarrow{{\mathrm{r}}_{\mathrm{CT}}}$ and $\overrightarrow{{\mathrm{r}}_{\mathrm{CBCT}}}$ can be related as:

4 $$\overrightarrow{{\mathrm{r}}_{\mathrm{CT}}}=R\cdot \overrightarrow{{\mathrm{r}}_{\mathrm{CBCT}}}+ \overrightarrow{T},$$

where ${R}$ is the rotational matrix that performs the intrinsic rotation as described above, represented in the basis vectors {$\widehat{{{e}}_{{x}}},\widehat{{{e}}_{{y}}},\widehat{{{e}}_{{z}}}$} of the linac coordinate system; $\overrightarrow{{T}}$ is the translation vector. When expressed with respect to the basis {$\widehat{{{e}}_{{x}}},\widehat{{{e}}_{{y}}},\widehat{{{e}}_{{z}}}$}, $\overrightarrow{{T}}$ can be written as:

5 $$\vec{T} = { }R \cdot \left( {\begin{array}{*{20}c} {{\text{Lat}}} \\ {{\text{Vrt}}} \\ {{\text{Lng}}} \\ \end{array} } \right).$$

Any intrinsic rotation can be transformed to an extrinsic rotation by the same angles but with inverted order of elemental rotations, so ${R}$ can be calculated as:

6 $$\begin{aligned} R = & R_{y} \cdot R_{x\prime } \cdot R_{z\prime \prime } = R_{z} \cdot R_{x} \cdot R_{y} \\ = & \left( {\begin{array}{*{20}c} {\cos \psi } & 0 & {\sin \psi } \\ 0 & 1 & 0 \\ { - \sin \psi } & 0 & {\cos \psi } \\ \end{array} } \right) \cdot \left( {\begin{array}{*{20}c} 1 & 0 & 0 \\ 0 & {\cos \theta } & { - \sin \theta } \\ 0 & {\sin \theta } & {\cos \theta } \\ \end{array} } \right) \cdot \left( {\begin{array}{*{20}c} {\cos \varphi } & { - \sin \varphi } & 0 \\ {\sin \varphi } & {\cos \varphi } & 0 \\ 0 & 0 & 1 \\ \end{array} } \right) \\ = & \left( {\begin{array}{*{20}c} {\sin\varphi \sin\theta\sin\psi+\cos\varphi\cos\psi} & {\cos\varphi\sin\theta\sin\psi-\sin\varphi\cos\psi} & {\cos\theta\sin\psi} \\ {\sin\varphi\cos\theta} & {\cos\varphi\cos\theta} & {-\sin\theta} \\ {\sin\varphi\sin\theta\cos\psi-\cos\varphi\sin\psi} & {\cos\varphi\sin\theta\cos\psi+\sin\varphi\sin\psi} & {\cos\theta\cos\psi} \\ \end{array} } \right) \\ \end{aligned}$$

The target shift vector $\overrightarrow{\Delta {r}}$ is:

7 $$\overrightarrow{\Delta r}= \overrightarrow{{\mathrm{r}}_{\mathrm{CBCT}}}-\overrightarrow{{\mathrm{r}}_{\mathrm{CT}}}=\left({R}^{-1}-I\right)\cdot \overrightarrow{{\mathrm{r}}_{\mathrm{CT}}}-{R}^{-1}\cdot \overrightarrow{T},$$

where ${I}$ is the identity matrix. Under small angle approximation, ${{{R}}}^{-1}$ can be estimated as:

8 $${R}^{-1}={R}^{\mathrm{T}}\approx \left(\begin{array}{ccc}1& \varphi & -\psi \\ -\varphi & 1& \theta \\ \psi & -\theta & 1\end{array}\right).$$

Plugging Eq. (5) and (8) into Eq. (7) yields Eq. (1), i.e.,

9 $$\overrightarrow{\Delta r}=\left(\begin{array}{c}\Delta x\\ \Delta y\\ \Delta z\end{array}\right)\approx \left(\begin{array}{ccc}0& \varphi & -\psi \\ -\varphi & 0& \theta \\ \psi & -\theta & 0\end{array}\right)\cdot \overrightarrow{{\mathrm{r}}_{\mathrm{CT}}}-\left(\begin{array}{c}\mathrm{Lat}\\ \mathrm{Vrt}\\ \mathrm{Lng}\end{array}\right),$$

### B: Statistics of target volumes and dose quality indexes of Hyperarc plans

See Table 5.

**Table 5:** Statistics of target volumes and dose quality indexes of the original plan without intra-fractional movements

PTV margin	Quantity	Target volume (cc)	RTOG CI	RTOG HI	GI (*V*_50%PD_/*V*_100%PD_)
0 mm	Median	0.23	1.46	1.30	5.13
	SD	2.30	0.28	0.06	4.66
1 mm	Median	0.53	1.24	1.35	4.13
	SD	3.10	0.13	0.07	2.37
2 mm	Median	0.95	1.19	1.33	4.17
	SD	3.89	0.12	0.06	2.12

CI, HI and GI stand for conformity index, homogeneity index and gradient index respectively
